# Optimization of a Continuous Hybrid Impeller Mixer via Computational Fluid Dynamics

**DOI:** 10.1155/2014/619474

**Published:** 2014-07-13

**Authors:** N. Othman, S. K. Kamarudin, M. S. Takriff, M. I. Rosli, E. M. F. Engku Chik, M. A. K. Meor Adnan

**Affiliations:** ^1^Department of Chemical and Process Engineering, Faculty of Engineering and Built Environment, Universiti Kebangsaan Malaysia (UKM), 43600 Bangi, Selangor, Malaysia; ^2^Industrial Technology Division, Malaysian Nuclear Agency, 43000 Kajang, Selangor, Malaysia

## Abstract

This paper presents the preliminary steps required for conducting experiments to obtain the optimal operating conditions of a hybrid impeller mixer and to determine the residence time distribution (RTD) using computational fluid dynamics (CFD). In this paper, impeller speed and clearance parameters are examined. The hybrid impeller mixer consists of a single Rushton turbine mounted above a single pitched blade turbine (PBT). Four impeller speeds, 50, 100, 150, and 200 rpm, and four impeller clearances, 25, 50, 75, and 100 mm, were the operation variables used in this study. CFD was utilized to initially screen the parameter ranges to reduce the number of actual experiments needed. Afterward, the residence time distribution (RTD) was determined using the respective parameters. Finally, the Fluent-predicted RTD and the experimentally measured RTD were compared. The CFD investigations revealed that an impeller speed of 50 rpm and an impeller clearance of 25 mm were not viable for experimental investigations and were thus eliminated from further analyses. The determination of RTD using a *k*-*ε* turbulence model was performed using CFD techniques. The multiple reference frame (MRF) was implemented and a steady state was initially achieved followed by a transient condition for RTD determination.

## 1. Introduction

Mixing processes are widely used in industry for many applications, including homogenization of viscous complex liquids for polymer blending, paints, and solution polymerization [1]. Moreover, mechanically stirred vessels are widely used for mixing single-phase flows, blending homogenous liquids, such as lube oils and gasoline additives, and dilutions in chemical and mineral processing, wastewater treatment, and other industries [2]. To achieve the highest product quality and lowest production costs, mixing process efficiency and optimization are the main parameters to be addressed [3]. Nevertheless, if the spacing between the impellers is decreased to one-third of the tank diameter, the region between the turbines behaves anisotropically with the merging or diverging of flow [2]. Thus, if the distance between the impellers is significant, the fluid profile produces individual impeller profiles showing no interaction between the impellers. Recently, CFD techniques have been employed to provide detailed data on the flow field, which are impossible to obtain via experimentation. To model the flow behavior, the Reynolds-averaged Navier-Stokes (RANS) equations and a model of the Reynolds stresses with an appropriate turbulence model have been adopted [4]. To account for impeller revolutions in CFD simulations, sliding mesh (SM) and MRF techniques have been widely utilized [5–7].

In this study, the determination of RTD was performed by using a hybrid impeller consisting of Rushton and pitched blade impellers in a continuous mixer. The hybrid impeller design was based on the discussion by Sahle-Demessie et al. (2003), in which the use of axial or mixed impellers was reported to improve the flow profile by narrowing the RTD curves, creating a high Reynolds number, and avoiding back mixing [8]. To date, no studies have been conducted for dual integrated impeller mixers using both a Rushton turbine and a PBT. Moreover, the combination of axial/radial impellers, such as PBT/Rushton turbines, in a mixer will lead to significant improvement of the axial mixing compared to using Rushton turbines alone [9]. The purpose of this study is to investigate the effective parameters using a CFD approach to optimize the mixing efficiency of a hybrid mixer. Therefore, three main objectives will be covered in this paper. Initially, CFD was used to screen the parameter ranges to reduce the number of actual experiments needed. Second, the residence time distribution (RTD) was determined using the pulse method. Finally, the Fluent-predicted RTD and the experimentally measured value were compared.

## 2. Experimental

### 2.1. Stirred Vessel Configurations

**Figure 1:**
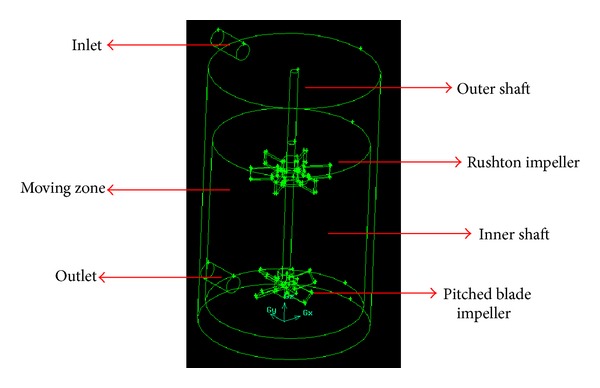
Reactor geometry of 3D simulation.

In this study, GAMBIT 2.4.6 and Fluent 6 software were used to draw the geometry of the object and to process the data, respectively. A model of the stirred tank shown in Figure 1 was constructed using 3D simulation. A dual impeller, composed of a standard 60° six-bladed Rushton turbine and a 90° four-bladed pitched blade turbine (PBT), was used to determine the RTD of the mixer. In this study, the Rushton turbine was mounted above the PBT. The speed of the impeller was varied at 50, 100, 150, and 200 rpm, and the height of clearance was varied at 25, 50, 75, and 100 mm. The dimensions of the hybrid mixer are given in Table 1.

### 2.2. Modeling and Numerical Aspects

Single-phase steady state CFD simulations were performed, the results of which were postprocessed to determine the effects of mixing speed and clearance on the mixing process. The standard* k*-*ɛ* model, the most widely used two-equation eddy viscosity model, was implemented for modeling the turbulence in the stirred tank reactor [4]. Better agreement with the experimental data was found for the standard *k*-*ε* model than the RNG model. Moreover, stability problems were experienced during tracer introduction when using the RNG model [10]. For simulating the flows generated by the impeller, the multiple reference frame (MRF) method was adopted [7]. This method allows for efficient steady state flow simulation in a mixer with a rotating impeller. Moreover, MRF is also considered to be an adequate and quasi-steady state approach [11]. To model the rotation of the impeller, the entire vessel was divided into two regions: the moving zone and the tank volume. An arbitrary moving zone was created around the impeller and inner shaft. By adopting this approach, the effects of the blade rotations were recorded per the reference frame. The convection term in the governing equation was modeled by a first-order scheme, and the SIMPLE algorithm was used to resolve the coupling between the velocity and the pressure parameters [7]. A 10^−4^ convergence limit was set for the solution. In this study, the boundaries for the inlet, the outlet, and the moving zone were assigned as velocity inlet, pressure outlet, and continuum, respectively.

### 2.3. Mesh Independence Test

**Figure 2:**
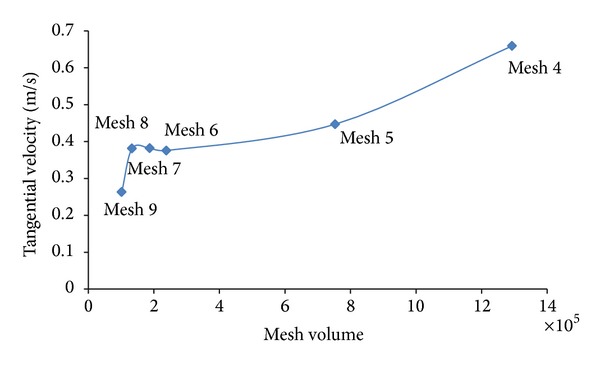
Mesh size determination.

After conducting the CFD analysis for different mesh size settings (4 to 9), the following results were observed for the tangential velocity. The results from Figure 2 clearly show that the CFD analysis produced a constant value for the tangential velocity at mesh settings of 6, 7, and 8. This observation indicates that the results will not be affected at these mesh values. For that reason, the mesh grid was fixed at 8 to save computational speed. The results also showed that the mesh volume was higher at smaller mesh settings of 4 and 5. Nevertheless, the system was unable to operate at mesh sizes smaller than 4 because of computational ability.

Tet/hybrid with type TGrid mesh was used throughout the study to mesh the tank domain for an efficient mesh resolution. An interval size of 8 was used for both the tank zone and the moving zone to accommodate the complex impeller geometry. There were approximately 100,905 computational cells for the moving zone and 81,382 computational cells for the tank domain.

### 2.4. Measurement of RTD

Upon determination of the converged profile of steady state flow, the model was switched to an unsteady state flow. The pulse method was adopted, which is similar to the method applied in the experiment. The introduction of the tracer was modeled using a two-species transport model. In this case, the species were water, which was present in bulk, and the injected tracer, which has similar properties as water. For the unsteady simulation using the pulse method, the initial condition had a filled tank with zero concentration of the tracer, followed by an injection of 1.0 concentration of tracer. Afterward, the tracer mass fraction was switched to 0 for data monitoring and collection. The collected data from the outlet were monitored and plotted to obtain the tracer concentration as a function of time.

### 2.5. Validation

The experiments were conducted with two different types of impellers attached to a common shaft: a Rushton turbine and a pitched blade turbine (PBT) for the radial and axial impellers, respectively. In this study, the Rushton turbine was mounted above the PBT, as illustrated in Figure 1. The sodium iodide detectors were positioned at the inlet and outlet and were connected to a data acquisition system (DAS). The DAS monitored the movement of the injected Tc-99m in the mixer during operation. Approximately 10 *μ*Ci of Tc-99m was used for each run. The Tc-99m was diluted to ensure that the radiation exposure was minimal, thus reducing the radiation risk to the operator. Prior to beginning the experimental work, the tank was loaded with tap water and the mixer was switched on until it reached a stable state. The experimental results were then compared with the obtained RTD values using CFD for verification.

## 3. Results and Discussion

### 3.1. Qualitative Analysis: Screening of Parameters

**Figure 3:**
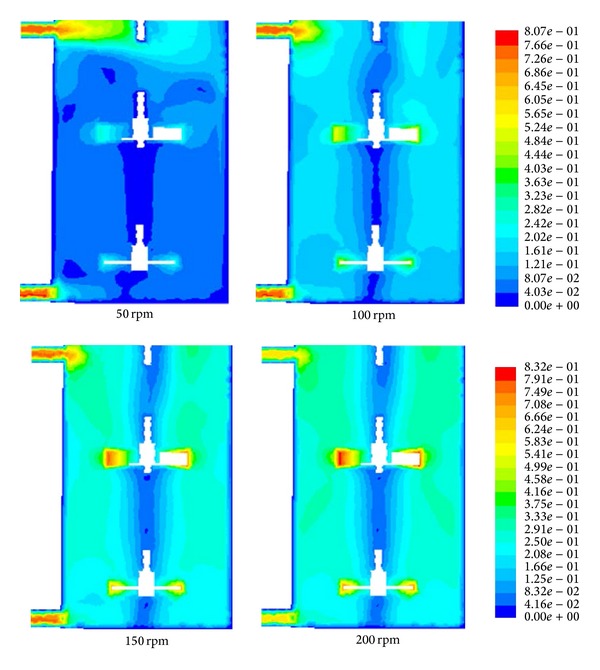
Velocity magnitude profile at various speeds.

**Figure 4:**
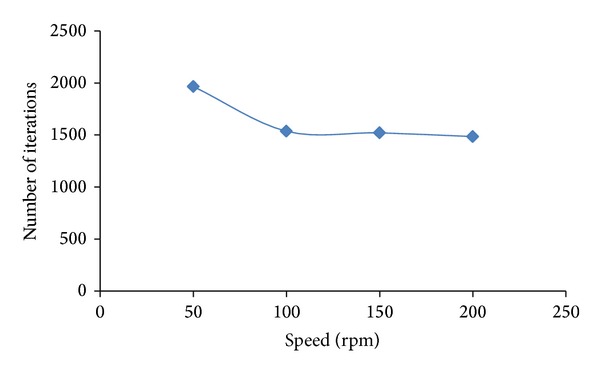
Effect of impeller speed on converged solution.

The screening of parameters, including impeller speed and clearance height, was observed.  Figure 3 shows the effect of speed variation on the flow field. In this paper, the clearance was fixed at 50 mm. It can be observed that, at 50 rpm, the flow field exhibits a nonhomogenous liquid with a low velocity magnitude identified at the tip of the individual impeller. The presence of an untouched zone or a dead zone is clearly observed, especially at the outer portion of the impeller where dark blue dominates the flow field. The dark blue diminishes slowly as the speed increases to 100 rpm, whereby a stronger velocity is observed at the tip of an individual blade. The increase of the speed to 150 and 200 rpm enables the effective dispersion of liquid in the mixer as shown in Figures 3(c) and 3(d), respectively. The flow field also exhibits a stronger circulation pattern extending over the large volume of the vessel. Moreover, the flow leaving the impeller in the radial direction and the subsequent entrainment flow into the outflow of the impeller. The flow also divides at the vessel wall and recirculates either directly into the impeller or as subsequent entrainment flow into the outflow of the impeller [4]. Additionally, the flow field below the impeller has a positive tangential velocity component similar to the radial-tangential jet, which originates from the impeller. The flow also tends to be directed toward the vessel wall where recirculation occurs, thus improving the mixing efficiency. Therefore, from these observations, a speed of 50 rpm was eliminated to accelerate the optimization because the dead volume at this speed is significant compared to the other speeds tested. The liquid is homogenously mixed for all ranges of speeds except at 50 rpm. The dead zone is subsequently reduced as the impeller speed increases. Moreover, the 50 rpm speed should be eliminated because the time needed for the solution to converge is longer compared to that at other speeds, which places a burden on the computational hardware, as shown in Figure 4.

The effects of various clearance heights on flow configuration are clearly described in Table 2, in which the impeller speed was fixed at 100 rpm. The flow configurations can be described by defining the lower impeller clearance, *C*, and the impeller spacing, *S*, both of which are normalized with the tank diameter, *T*. In this study, the value of *S*/*T* was 0.7. Therefore, all ranges of clearances showed parallel flow except for a clearance of 25 mm. According to Montante and Magelli (2004), critical impeller arrangements can lead to parallel, merging, or diverging flow profiles [2]. Homogenization in mixing operations is best practiced when the flow field formed from each impeller is wide enough so that there is no interaction between adjacent impellers. Moreover, the significant distance will ensure that the flow pattern produced by each impeller is similar to that determined by a single impeller. Thus, a clearance of 25 mm was eliminated because it produced a diverging type of flow.

### 3.2. RTD Determination Using CFD

**Figure 5:**
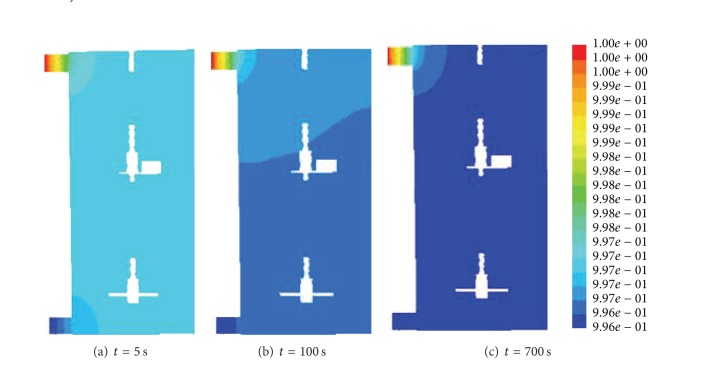
Concentration profile of the tracer under transient conditions.

The simulation of RTD was performed using the pulse method, which resembled the experimental approach. Under steady state conditions, a single-species flow field was obtained. Under transient conditions, the tracer was introduced as a momentary pulse at the feed entrance.  Figure 5 shows the concentration profile evolution of the tracer movement after injection into the mixer.  Figure 5(a) shows the distribution of the tracer after its introduction into the mixer until it gradually occupied the mixer (Figure 5(b)) and was well distributed (Figure 5(c)) at the indicated times.

### 3.3. Effects of Parameters on RTD

**Figure 6:**
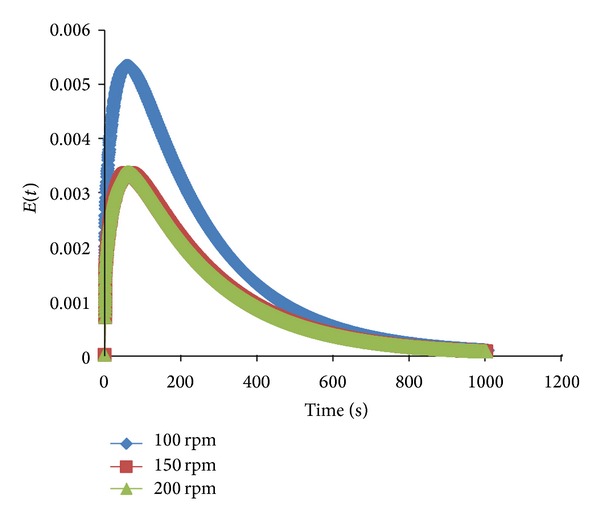
Effects of impeller speed on RTD.

Figure 6 shows that varying the impeller speed at 100, 150, and 200 rpm had a significant effect on the hybrid impeller mixer. In this study, the clearance was fixed at 50 mm. The RTD curve was narrow, with a sharp peak obtained at an impeller speed of 100 rpm, whereas at higher impeller speeds the RTD curves were shorter and flatter. This result may be because the presence of radial Rushton turbines generates a very strong radially outward flow, whereas the axial turbine (PBT) generates a separate circulation loop within the reactor. Moreover, the Rushton turbine is able to produce two symmetrical loops on the top and bottom of the impeller, whereas the PBT produces a pair of circulation loops on its side. Thus, the collision from the circulation loops of the respective turbines improves the mixing process.

**Figure 7:**
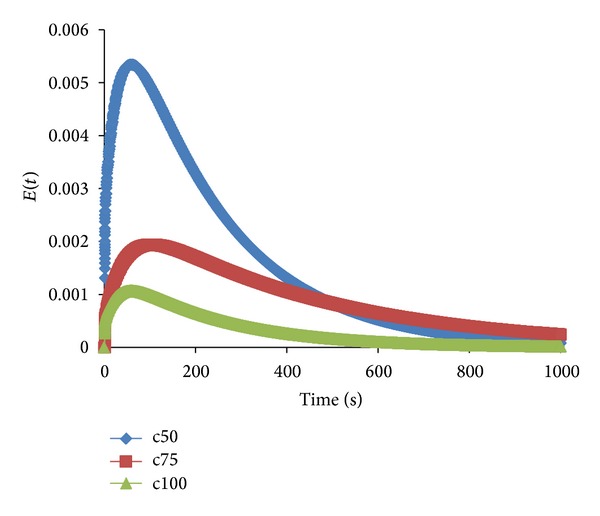
Effects of clearance height on RTD.

Moreover, the effects of clearance can be observed in Figure 7. The clearance of the impeller was varied at 50, 75, and 100 mm from the tank floor. Figure 7 shows that, as the clearance increases from 50 mm to 100 mm, the RTD curves gradually widen. At 50 mm, the RTD curve produces a sharp peak that is narrower than the others. The ability of the impeller to sweep the base of the tank was disturbed when the clearance was reduced to 50 mm. This situation produced a positive tangential velocity below the impeller. After flowing vertically along the wall, the fluid will have a recirculation flow pattern toward the axis of the tank. This component is similar to the radial-tangential jet that originates from the impeller [4]. Thus, it can be concluded that, by using a hybrid impeller mixer, the optimum conditions for the RTD curve are an impeller speed of 100 rpm and a clearance height of 50 mm.

### 3.4. Validation

**Figure 8:**
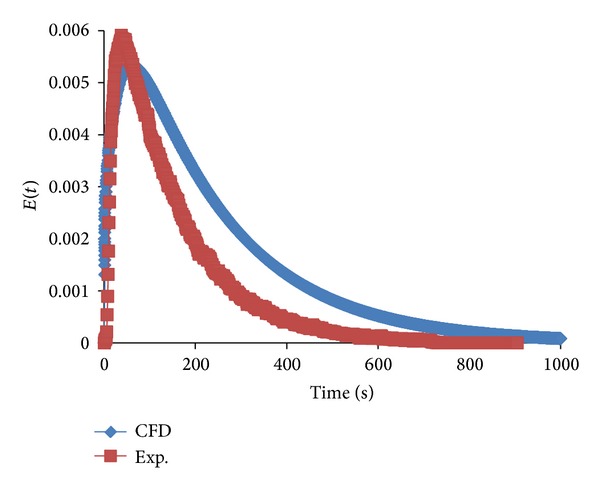
Comparison of simulated RTD with experimental data.

In the present work, CFD results showed that the optimum conditions for good RTD curves are an impeller speed of 100 rpm and a clearance height of 50 mm. A comparison of the Fluent-predicted RTD and the radiotracer experiment is shown in Figure 8. The prediction shows a similar curve although it does not overlap with the experimental curve. Thus, it can be observed that the CFD results slightly overpredicted the experimental measurements. In a real stirred tank reactor, large vortices may be present, resulting in macrostabilities. This phenomenon promotes tracer mass exchange through this boundary [5]. Nevertheless, CFD codes cannot fully account for this condition. As a result, overprediction of the RTD was obtained. Moreover, in the future, another turbulence model will be employed using the RNG k-*ε* model or the Reynolds stress model (RSM).

## 4. Conclusion

In this study, it can be concluded that CFD with an MRF can successfully assist in screening the effective parameters for mixing efficiency. The ranges of recommended speeds to accelerate optimization are 100, 150, and 200 rpm, and the ranges of recommended clearance heights are 50, 75, and 100 mm for hybrid impellers in mixing vessels. These parameter ranges will be used in future experiments to optimize the mixing process. CFD investigations have shown that an impeller speed of 50 rpm and an impeller clearance of 25 mm were not viable for experimental investigations and were thus eliminated from further analysis. The effect of impeller speed showed that the RTD curve was narrower at 100 rpm compared to other speeds. Moreover, significant differences were also observed when the clearance was reduced from 100 mm to 50 mm. This study showed that the optimum conditions for mixing operations in a hybrid impeller are 100 rpm and 50 mm for impeller speed and clearance height, respectively, which resulted in an RTD curve with a strong, sharp peak. Finally, the model prediction of RTD was compared with the experimental RTD under the optimum conditions. It was found that the predicted model slightly overpredicted the experimental measurements. One of the main reasons for this discrepancy is the effect of turbulence parameters on the predicted model. Future work in this area will employ the RNG* k*-*ε* model or the Reynolds stress model (RSM) instead of the standard *k*-*ε* model.

## Figures and Tables

**Table 1:** Dimensions of the hybrid impeller mixer.

	Descriptions	Dimensions (mm)
Impeller	Blade height Blade width Blade thickness	16 34 3

Tank	Height Diameter	350 218

Inlet/outlet	Diameter	21

Moving zone	Height Diameter	400 200

**Table 2:** Effects of various clearance heights on the type of flow.

Clearance (mm)	*C*/*T*	*S*/*T*	Type of flow
25	0.12	0.7	Diverging
50	0.24	0.7	Parallel
75	0.36	0.7	Parallel
100	0.48	0.7	Parallel
